# NeuroStream: an interactive platform for exploratory visualization and harmonization of multicohort brain MRI data

**DOI:** 10.1093/bioadv/vbag117

**Published:** 2026-04-26

**Authors:** Myeongji Cho, Byung Soo Park, Hye Ryeong Nam, Jae Pil Jeon, Sang Cheol Kim

**Affiliations:** Division of Healthcare and Artificial Intelligence, Department of Precision Medicine, National Institute of Health, Korea Disease Control and Prevention Agency, Cheongju-si 28159, Republic of Korea; Division of Healthcare and Artificial Intelligence, Department of Precision Medicine, National Institute of Health, Korea Disease Control and Prevention Agency, Cheongju-si 28159, Republic of Korea; Division of Healthcare and Artificial Intelligence, Department of Precision Medicine, National Institute of Health, Korea Disease Control and Prevention Agency, Cheongju-si 28159, Republic of Korea; Department of Precision Medicine, National Institute of Health, Korea Disease Control and Prevention Agency, Cheongju-si 28159, Republic of Korea; Division of Healthcare and Artificial Intelligence, Department of Precision Medicine, National Institute of Health, Korea Disease Control and Prevention Agency, Cheongju-si 28159, Republic of Korea

## Abstract

**Motivation:**

Large-scale neuroimaging studies increasingly integrate brain MRI data from multiple cohorts and acquisition sites. Exploratory visualization and harmonization are essential for identifying batch effects, assessing preprocessing strategies, and preserving biologically meaningful variation. However, existing tools are often cohort-specific, rely on static visualizations, or separate harmonization from data exploration.

**Results:**

We present NeuroStream, an interactive application for real-time visualization and harmonization of multicohort brain MRI quantitative data. Built using the Streamlit framework, NeuroStream enables the exploration of quantitative imaging features derived from structural MRI along with demographic and clinical variables. The platform supports preprocessing and harmonization options, including log transformation, intracranial volume normalization, and ComBat-based batch correction, which can be compared interactively using dynamic visualizations. NeuroStream provides principal component analysis, distributional plots, and group comparison statistics. In addition, the platform supports covariate-adjusted regression-based evaluation of site effects and residual diagnostics, enabling quantitative and visual assessment of cohort-related variability across preprocessing settings. All visualizations presented in this manuscript were generated using transformed example datasets designed to illustrate the functionality of the platform, rather than to report original subject-level measurements, while NeuroStream itself supports direct analysis of user-provided tabular data. Using transformed example datasets derived from BICWALZS and KoGES multicohort MRI data, we demonstrate intuitive inspection of batch-related variance and harmonization outcomes without custom scripting.

**Availability and implementation:**

NeuroStream is implemented in Python and freely available at https://github.com/NIHxAI/NeuroStream.

## 1 Introduction

Harmonization and visualization are critical components of multicohort neuroimaging research. As large-scale MRI studies increasingly integrate quantitative imaging features with demographic and clinical variables across cohorts, the variability introduced by acquisition sites, scanners, and preprocessing pipelines can obscure biologically meaningful signals. Therefore, transparent visualization is essential for assessing data comparability and interpreting harmonization outcomes (Fortin *et al*. 2017).

Various tools have been developed for MRI visualization and analysis; however, most focus on image-level inspection, quality control, or the execution of standardized analysis pipelines. While widely used viewers such as FSLeyes, distributed as part of the FSL software suite, facilitate intuitive exploration of volumetric MRI data (Jenkinson *et al*. 2012), container-based platforms such as NeuroDesk enhance the reproducibility of multisite workflows (Renton *et al*. 2024). However, these tools provide limited support for the interactive comparison of quantitative features across datasets or for the visual assessment of cohort-related variability. In particular, few existing applications allow researchers to iteratively evaluate preprocessing and harmonization strategies while directly inspecting their effects on data distributions and multivariate structure (Rue-Albrecht *et al*. 2018, Benedetti *et al*. 2025).

To address these limitation, we developed NeuroStream, an interactive platform that integrates exploratory visualization with harmonization workflows for multicohort brain MRI quantitative data. NeuroStream enables the real-time inspection of quantitative imaging feature distributions, assessment of cohort-driven variance, and validation of harmonization outcomes using dynamic graphics and statistical summaries, thus supporting the transparent and reproducible analysis of harmonized neuroimaging datasets.

## 2 Methods

### 2.1 Data sources

To demonstrate the functionality of NeuroStream, data from two large-scale multicenter cohorts were used: the Biobank Innovations for Chronic Cerebrovascular Disease with Alzheimer’s Disease Study (BICWALZS) and the Korean Genome and Epidemiology Study (KoGES). From each cohort, a representative subset of approximately 500 participants was randomly selected for analysis. Each dataset included demographic variables (e.g. age and sex), laboratory parameters, and clinical phenotypes. Structural MRI scans were provided as a DICOM series and underwent deidentification prior to analysis. From the full set of MRI-derived quantitative measures, a curated subset of 66 representative regional brain volume features was selected to support interactive visualization and exploratory analyses. These volumetric features were extracted using Neurophet AQUA. For the purpose of demonstrating the visualization workflow, all MRI-derived features used in this study were transformed so that original subject-level volumetric values were not directly represented, while preserving distributional structure and relative patterns relevant for exploratory visualization. These transformations were applied solely to generate illustrative example data for the manuscript figures and do not reflect limitations of the NeuroStream platform itself.

### 2.2 Input data requirements

NeuroStream accepts tabular datasets in CSV or TSV format, where rows correspond to subjects and columns correspond to imaging-derived features. A designated batch variable (e.g. cohort or site) may contain an arbitrary number of levels and is treated as a categorical factor in regression-based analyses and harmonization procedures. Additional covariate columns (e.g. age, sex, education level, disease status, or other user-specified variables) can be specified by the user for covariate-adjusted regression models and ComBat harmonization.

### 2.3 Data handling and preprocessing

NeuroStream was implemented in Python using Streamlit as the user interface and Plotly for interactive visualization. Users can upload tabular datasets containing imaging-derived features, along with demographic and clinical variables. The uploaded files are automatically converted to the Feather format to support efficient caching, reproducibility, and fast input/output operations.

The application provides modular preprocessing and harmonization options that can be applied interactively:

- Log transformation: log1p transformation to reduce right-skewed distributions, with an optional subsequent log transformation followed by z-score standardization.
- Z-score standardization: feature-wise scaling to zero mean and unit variance.
- Intracranial volume (ICV) normalization: volumetric measures divided by subject-specific ICV to adjust for head size effects.
- Outlier trimming: internal trimming of values outside the 0.1–99.9 percentile range.
- ComBat harmonization: batch effect correction across cohorts using pyComBat, with user-specified biological covariates (e.g. age, sex, disease status, or other user-specified variables) included in the design matrix (Johnson *et al*. 2007, Fortin *et al*. 2017).

To avoid potential overcorrection, ICV normalization and ComBat harmonization are evaluated separately by default, with their combined application available as a sensitivity analysis.

### 2.4 Visualization and statistical summaries

Descriptive statistics, including the mean, median, standard deviation, and sample size, are computed for each variable. Distributional differences across cohorts and preprocessing conditions are visualized using boxplots and violin plots, with preprocessing states clearly indicated in the visualization context (e.g. plot titles or interface selections).

Group differences are assessed using independent t-tests for binary grouping variables. For multi-level grouping variables (e.g. age group or education level), pairwise *t*-tests are provided as descriptive summaries to facilitate exploratory comparison of group means. To further evaluate site-related variation, NeuroStream implements covariate-adjusted regression models in which ROI values are modeled as a function of site and biological covariates, and omnibus *F*-statistics and *P* values are computed from these models for each ROI. Associations between continuous variables are explored using scatter plots with groupwise trend lines to visually convey the overall patterns (Cleveland 1979). Multivariate structures and cohort-related batch effects are examined using incremental principal component analysis (PCA), with separate visualizations generated for raw, ICV-normalized, and ComBat-harmonized datasets.

### 2.5 Covariate-adjusted evaluation of site effects

To formally evaluate batch (site) effects while accounting for biological covariates, ROI-wise linear regression models are implemented within NeuroStream.

For each imaging-derived feature, the following model is fitted:


$$\mathrm{ROI}=\beta_{0}+{X_{\mathrm{site}}\beta }_{\mathrm{site}}+X_{\mathrm{covariates}}\beta_{\mathrm{covariates}}+\epsilon$$

where site was treated as a categorical fixed effect and covariates included age, sex, and other user-specified variables. Omnibus *F*-statistics and *P* values were computed from these covariate-adjusted regression models for each ROI and visualized within the NeuroStream interface to enable quantitative comparison of site-related variation.

To further distinguish biological variation from residual batch-related structure, covariate-adjusted residuals were computed using a covariate-only model that excluded the site factor:


$$\mathrm{ROI}=\beta_{0}+\beta_{\mathrm{covariates}}+\epsilon$$

Residuals were visualized across sites to examine whether site-dependent structure persisted after removing expected biological effects.

Both regression-based inference and residual diagnostics can be applied to raw and harmonized datasets to assess harmonization effectiveness. This two-step framework combining regression-based inference and residual diagnostics enables quantitative and visual evaluation of site effects before and after harmonization.

### 2.6 Data privacy and deployment

NeuroStream does not require raw imaging files (e.g. DICOM or NIfTI). NeuroStream is intended for de-identified tabular inputs and does not require personally identifiable information for analysis.

NeuroStream is distributed as an open-source software package and is executed locally on the user’s machine via a Streamlit-based local server. As a result, uploaded datasets remain within the user’s computing environment and are not transmitted to external servers.

For institutions with strict data governance policies, NeuroStream can therefore be run entirely within secure institutional infrastructures. The example dataset distributed with the NeuroStream repository is a synthetic toy dataset provided solely to illustrate the expected input data structure and does not contain real cohort data.

## 3 Results

### 3.1 Performance and usability

Using the Apache Arrow Feather format improved the data-loading speed by approximately threefold compared with CSV-based workflows, enabling smooth interaction for datasets comprising several thousand participants. Modular preprocessing ensured that changes in normalization or harmonization settings were immediately reflected across all visualizations.

**Figure 1:**
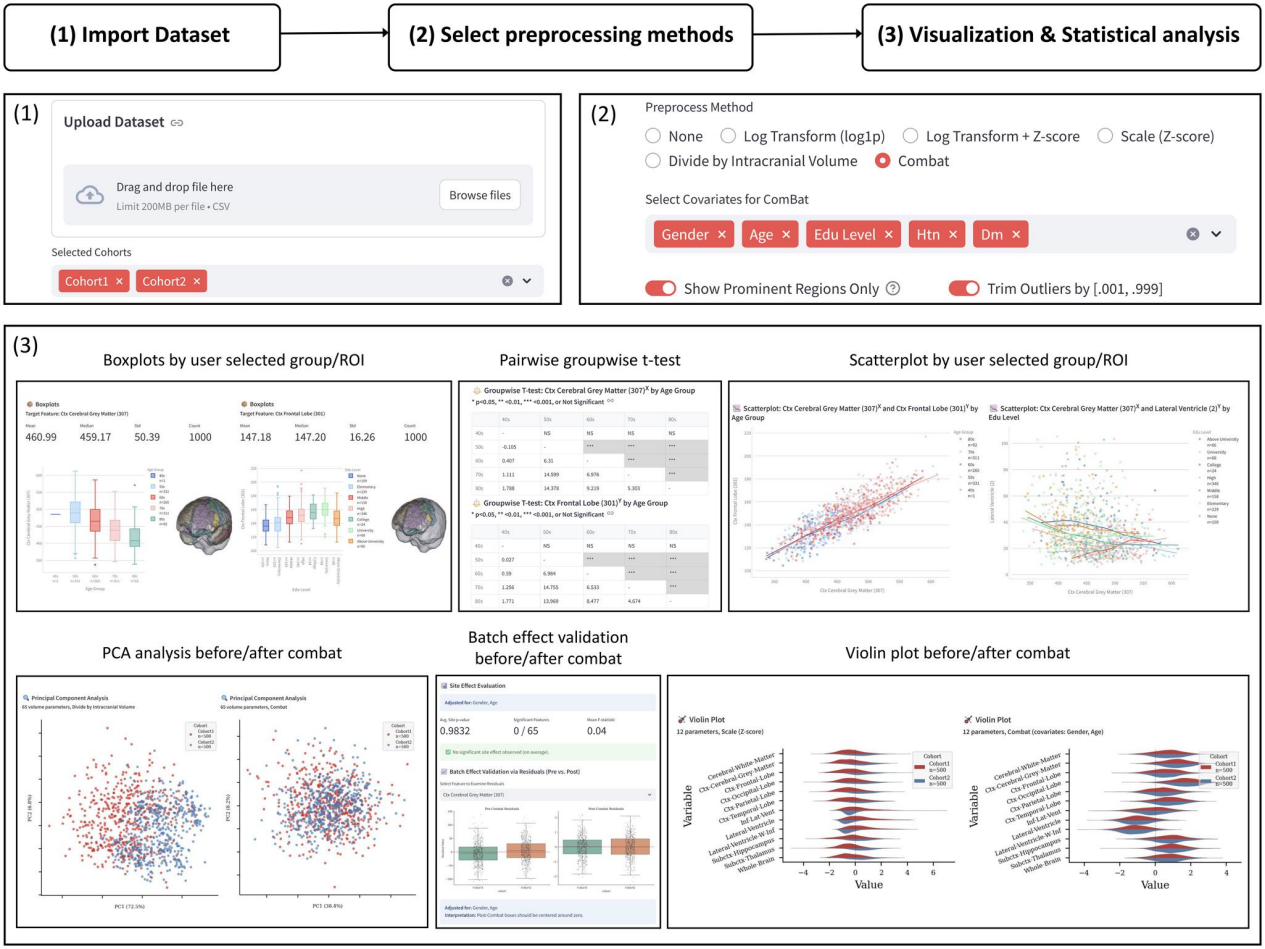
Interactive three-step workflow of NeuroStream for visualization and harmonization of multicohort brain MRI quantitative data. (i) Dataset import. Users upload tabular datasets containing MRI-derived quantitative features along with associated demographic and clinical variables. Multiple cohorts can be selected simultaneously, and the uploaded files are automatically aligned based on shared variables to enable integrated exploratory analysis. (ii) Selection of preprocessing and harmonization methods. NeuroStream provides modular preprocessing options, including log transformation (log1p), *z*-score scaling, intracranial volume (ICV) normalization, and ComBat-based batch effect correction. Users may specify biological covariates (e.g. age, sex, or other user-defined variables) in the harmonization model. Additional controls allow users to restrict visualization to selected brain regions and to trim extreme values to facilitate distributional inspection. (iii) Visualization and statistical analysis. The application generates interactive visual outputs, including boxplots and violin plots for distributional comparisons, scatter plots with groupwise trend lines for continuous associations, pairwise t-test summaries, and principal component analysis (PCA) visualizations before and after batch correction. NeuroStream also supports covariate-adjusted regression-based evaluation of site effects and residual-based diagnostics, enabling users to assess cohort-related variability and harmonization effectiveness in real time.

Figure 1 summarizes the interactive three-step workflow of NeuroStream, illustrating dataset import, selection of preprocessing and harmonization options, and real-time visualization of quantitative MRI features within a unified Streamlit-based application.

### 3.2 Interactive visualization and harmonization assessment

NeuroStream provides multiple visualization modes for exploratory analyses. Distribution plots highlighted cohort-specific shifts in regional brain volumes under raw and ICV-normalized conditions, whereas ComBat harmonization reduced these differences both visually and statistically, as demonstrated by attenuation of site-associated *F*-statistics and diminished residual site clustering. PCA-based visualizations further showed reduced cohort-driven separation following harmonization, supporting improved cross-site comparability.

### 3.3 Covariate-adjusted site effect evaluation

Prior to harmonization, covariate-adjusted regression models revealed substantial site-related variation across multiple regional brain volume features, as indicated by elevated omnibus *F*-statistics and low *P* values. Covariate-adjusted residual visualizations revealed persistent site-dependent structure even after removal of biological covariate effects.

Following ComBat harmonization, regression-based evaluation demonstrated marked attenuation of site-associated *F*-statistics across ROIs. The number of features exhibiting statistically significant site effects was substantially reduced relative to the raw dataset. Residual-based visualizations similarly showed diminished site-related clustering, indicating effective mitigation of batch-related variability.

Together, these findings demonstrate that NeuroStream enables both statistical and visual assessment of harmonization effectiveness.

### 3.4 Example application

Using multicohort volumetric MRI data, NeuroStream captures the expected age-related decreases in cortical gray matter and education level-related differences in frontal lobe volume. Scatter plots demonstrated preserved anatomical relationships, including positive correlations between cortical gray matter and frontal-lobe volumes and inverse associations between lateral ventricle and frontal lobe volumes across cohorts. These illustrative patterns are consistent with established clinical and epidemiological observations, supporting the use of NeuroStream for biologically plausible exploratory analysis of multicohort MRI data.

## 4 Conclusion

NeuroStream is an interactive visualization and harmonization framework for multicohort brain MRI quantitative data. By integrating preprocessing, batch correction, regression-based site-effect evaluation, and real-time visualization, the platform enables a transparent and reproducible assessment of cohort effects and harmonization outcomes without requiring custom scripting. While the current implementation focuses on cross-sectional ComBat harmonization of structural MRI-derived features, the framework is designed to be extensible. Future development will incorporate advanced ComBat-family methods, including longitudinal ComBat for repeated-measures data and CovBat for covariance harmonization, enabling more comprehensive evaluation of longitudinal and multivariate imaging datasets. Future extensions will also incorporate multivariate non-parametric testing to complement the current ROI-level regression-based diagnostics. In addition, NeuroStream provides a foundation for future integration of PET and functional MRI data within multimodal neuroimaging studies.

## Data Availability

The NeuroStream source code and documentation are publicly available at: https://github.com/NIHxAI/NeuroStream.

## References

[vbag117-B1] Benedetti G , Seraidarian E, Pralas T, Jeba A, Borman T, Lahti L. iSEEtree: interactive explorer for hierarchical data. Bioinform Adv 2025;5:vbaf107. 40406671 10.1093/bioadv/vbaf107 PMC12095132

[vbag117-B2] Cleveland WS. Robust locally weighted regression and smoothing scatterplots. J Am Stat Assoc 1979;74:829–36.

[vbag117-B3] Fortin JP , Parker D, Tunç B et al Harmonization of multi-site diffusion tensor imaging data. Neuroimage 2017;161:149–70. 28826946 10.1016/j.neuroimage.2017.08.047 PMC5736019

[vbag117-B4] Jenkinson M , Beckmann CF, Behrens TE et al Fsl. Neuroimage 2012;62:782–90. 21979382 10.1016/j.neuroimage.2011.09.015

[vbag117-B5] Johnson WE , Li C, Rabinovic A. Adjusting batch effects in microarray expression data using empirical bayes methods. Biostatistics 2007;8:118–27. 16632515 10.1093/biostatistics/kxj037

[vbag117-B6] Renton AI , Dao TT, Johnstone T et al Neurodesk: an accessible, flexible and portable data analysis environment for reproducible neuroimaging. Nat Methods 2024;21:804–8. 38191935 10.1038/s41592-023-02145-x PMC11180540

[vbag117-B7] Rue-Albrecht K , Marini F, Soneson C et al iSEE: interactive summarizedexperiment explorer. F1000Res 2018;7:741. 30002819 10.12688/f1000research.14966.1 PMC6013759

